# Autism spectrum disorder and personality disorders: How do clinicians carry out a differential diagnosis?

**DOI:** 10.1177/13623613231151356

**Published:** 2023-01-28

**Authors:** Clare S Allely, Emma Woodhouse, Raja AS Mukherjee

**Affiliations:** 1 University of Salford, UK; 2 King’s College London, UK; 3 Compass Psychology Services, UK; 4 Surrey and Borders Partnership NHS Foundation Trust, UK

**Keywords:** ASD, autism spectrum disorder, personality disorders

## Abstract

Although it is recognised that autism spectrum disorder (ASD) and personality disorders (PDs) have a variety of factors in common, the exact nature of the relationship between ASD and the PDs remains unclear. The overlapping symptom profiles and complexities in developmental trajectories in ASD and PDs can lead to diagnostic uncertainty and to challenges in determining differential diagnoses. Features of ASD and PD can be misattributed and easily lead to misdiagnosis or missed diagnoses in patients who have a diagnosis of ASD, which may impact therapeutic pathways. Since differentiating between ASD and PD is such a complex task with implications for therapeutic approaches, it has been argued that there is a need for additional understanding and markers for facilitating diagnostic procedures. There is an urgent need to explore how clinicians make diagnostic decisions and the challenges they face in forming diagnostic conclusions. Where overlaps exist, it is important to consider how clinicians interpret and attribute certain characteristics within the context of ASD and/or PD diagnoses.

**Lay Abstract:**

It is now recognised that autism spectrum disorder (ASD) and personality disorders (PDs) have a variety of factors in common. However, the exact nature of the relationship between ASD and the PDs remains unclear. The overlapping symptom profiles of ASD and PDs can lead to diagnostic uncertainty – features of ASD and PD can be misattributed and easily lead to misdiagnosis of ASD patients. Since differentiating between ASD and PD is such a complex task, it has been argued that there is a need for additional understanding and markers for facilitating diagnostic procedures. There is an urgent need to explore, first, how clinicians make diagnostic decisions and, second, how to effectively deal with the challenges and difficulties they face when making decisions. Also, where there are clear overlaps, how do clinicians choose how to attribute labels in order to understand the person.

## Existing research

Autism spectrum disorder (ASDs) is defined, in the fifth edition of the *Diagnostic and Statistical Manual of Mental Disorders* (*DSM*-5, American Psychiatric Association [APA], 2013) as persistent deficits in social communication and social interaction as well as restricted, repetitive pattern of behaviour, interests or activities which causes clinically significant impairment. Takara and colleagues (2015) have previously highlighted that ASD (a neurodevelopment disorder) is frequently overlooked or misdiagnosed in adult patients, particularly in those patients with psychiatric mental health comorbidities. Reasons for this may include insufficient training and experience among professionals in detecting ASD in adult patients and milder and/or atypical autistic traits compared with the more prominent symptoms of psychiatric comorbidities (Takara et al., 2015).

The exact nature of the relationship between ASD and the personality disorders (PDs) remains unclear (Lugnegård et al., 2012). For example, while there are different periods of developmental origin suggested, the impact of postnatal influences on early developmental trajectories is unknown. The *DSM*-5 specifies that PDs typically develop in adolescence to early adulthood while ASD starts in early childhood (see also Alafia & Manjula, 2020; Velikonja et al., 2019). There have been relatively few studies exploring personality traits or personality pathology in autistic people (e.g. Kanai et al., 2011; Ozonoff et al., 2005; Soderstrom et al., 2002). However, existing research indicates that patients who have a diagnosis of ASD exhibit a specific cluster of personality traits. Hofvander and colleagues (2009) found that 42 of 62 (68%) patients who had a diagnosis of ASD fulfilled *DSM* diagnostic criteria for at least one PD. Lugnegård and colleagues (2012) found 26 of 54 patients who had a diagnosis of ASD met the diagnostic criteria for at least one PD.

Surprisingly, there has been a lack of research exploring personality traits or personality pathology in ASD compared with specific PDs. Questions arise as to why this may be. For example, Hrdlicka and Dudova (2013) have suggested that there may be a developmental transition between ASD and PD.

## Overlaps between ASD and PD

Due to phenomenological similarities, it is recognised that ASD and PD have a variety of factors in common. The overlap between PDs and ASD is most evident in the realms of social communication and interaction. For example, both involve difficulties in developing and maintaining relationships and result in impairment across several domains of functioning (such as occupation, intimate relationships, friendships) (APA, 2013). Dudas and colleagues (2017) noted that the symptomatic overlap of ASD and PDs can result in differential diagnostic uncertainty most notably in women (Lugnegård et al., 2012). Clinical observations have indicated a number of similarities between ASD and borderline personality disorder (BPD) including identity problems, intense anger, self-damaging behaviour and severe problems in interpersonal relationships (e.g. Fitzgerald, 2005; Pelletier, 1998). BPD can mask autistic features but may also present as a comorbidity, which can lead to misdiagnosis (Takara et al., 2015).

Focusing on specific symptoms and characteristics without looking at the overall clinical picture may lead to misdiagnosis. For example, characteristics such as flat intonation, minimal changes in facial expression and reduced use of emotional gestures (all of which can be associated with ASD) could also be conceptualised as ‘emotional coldness, detachment, or flattened affectivity’, which is one of the criteria listed for Schizoid PD. This is one of many examples which could lead to misattribution of symptomology between ASD and PD.

## Differences between ASD and PD and approach to diagnostic assessment

While there are considerable overlaps in symptoms between ASD and certain PDs (Lugnegård et al., 2012), it is important to consider the differences in these presentations. These differences are crucial in the process of assessing differential diagnoses.

Both ASD and PD diagnoses require pervasive and persistent patterns of behaviour that lead to impairments in functioning. While there are some overlaps between specific characteristics of ASD and PD, it is important to consider the overall clinical presentation across the lifespan. This includes a specific observational assessment for ASD (such as the Autism Diagnostic Observation Schedule, ADOS-2; Lord et al., 2012) looking at the subtle nuances and clinical quality of current behaviours, more general observations from professionals involved in their care, a detailed developmental history (with input from a parent/family member wherever possible) and consideration of differential diagnoses within a multidisciplinary team.

The way in which particular symptoms and characteristics ‘cluster’ together may help clinicians to identify whether their presentation is better explained by ASD, PD, or both. For example, a diagnosis of ASD under *DSM*-5 and the *International Statistical Classification of Disease and Related Health Problems, 10th Revision* (ICD-10) requires the presence of restricted and repetitive behaviours and interests. This includes preoccupations, circumscribed interests, difficulties with minor changes to routine, ritualistic behaviours, stereotyped or repetitive motor movements, use of objects and speech sensory processing differences. While some restricted and repetitive behaviours may be observed within specific PD diagnoses (e.g., Obsessive Compulsive / Anankastic PD), these behaviours are not primary features of other PDs (such as Borderline, Antisocial or Schizoid PD). For individuals who have become ‘institutionalised’ by routines in inpatient and secure settings, it is essential to determine whether the insistence on routine predated their admission to that particular setting, and whether it has been a persistent feature of their presentation since early childhood. Before attributing individual characteristics to ASD or PD, it is important to consider the overall presentation, clusters of symptoms and developmental trajectory.

ASD and PD are clinical diagnoses which can inform therapeutic approaches, but it is important that the individual’s unique set of strengths, difficulties and needs are considered throughout clinical formulations and treatment plans. Tools such as the International Personality Disorder Examination (IPDE, Loranger, 1999; Loranger & Mombour, 1996), ADOS-2 and Autism Diagnostic Interview – Revised (ADI-R, Lord et al., 1994) can be a valuable part of the assessment process; it is important to be aware that they are intended to inform clinical thinking rather than provide a clear-cut ‘answer’ in relation to diagnoses. Qualitative information should be carefully considered and in some cases, clinical judgement may override quantitative scores and cutoffs.

Recently, Gordon and colleagues (2020) published guidelines for clinicians to help them differentiate between ASD and BPD. The guidelines are definitely a step forward. However, the authors note that they developed the guidelines based on the available theoretical literature and the authors’ experience in clinical practice rather than any empirical evidence. It is also important to stress that there is relatively little theoretical literature currently available. To our knowledge, there have been no empirical studies to date which have explored the inadequacies of current diagnostic/screening measures in differentiating between people with ASD (Kenny et al., 2016) and PD.

Since differentiating between ASD and PD is such a complex task, it has been argued that there is a need for additional markers for facilitating diagnostic procedures. Differentiating patients who have a diagnosis of ASD from patients with PDs is relevant for prognostic evaluation and decisions regarding appropriate treatment (Strunz et al., 2015).

Given the issues discussed above regarding the challenges surrounding diagnosing PDs and ASD (including misdiagnosis) there is a need to explore how these complexities are experienced by clinicians involved in diagnostic processes. Research can offer some insights that show how clinicians will act in a manner linked to diagnostic manuals. Even there, differences in interpretation will exist as has been seen in psychiatric research across the years. In other words, the evaluation or interpretation of an individual’s phenomenology often differs across professionals leading to multiple interpretations of phenomenology and presenting characteristics. Given this, case discussions take place frequently between different professionals in order to reach a diagnostic agreement. By understanding how clinicians currently do this (with respect to ASD and PDs), it will allow us to then begin to pull apart if the populations are the same and begin to better delineate the two.

## Summary

Understanding how clinicians approach situations where there are diagnostic complexities around ASD and/or PD and ensuring that professionals have access to appropriate training is essential. However, it is also important to consider putative factors, such as delineating innate differences in brain development from psychosocial difficulties. Finally, although we have focused on ASD and PD, there is also the need to look at common wider neurodevelopmental disorder overlaps with ASD such as attention-deficit/hyperactivity disorder (ADHD). Characteristics commonly associated with ADHD (such as impulsivity and emotional dysregulation) also overlap with symptoms of certain PDs, and when ASD and ADHD co-occur, this can further complicate the process of differential diagnoses.
